# Anti-EGFR-Conjugated Hollow Gold Nanospheres Enhance Radiocytotoxic Targeting of Cervical Cancer at Megavoltage Radiation Energies

**DOI:** 10.1186/s11671-015-0923-2

**Published:** 2015-05-15

**Authors:** Jiao Liu, Ying Liang, Ting Liu, Dengke Li, Xingsheng Yang

**Affiliations:** Department of Gynecology and Obstetrics, Qilu Hospital, Shandong University, Wenhuaxilu No. 107, Jinan, 250012 Shandong Province China; Department of General Surgery, Qilu Hospital, Shandong University, Wenhuaxilu No. 107, Jinan, 250012 Shandong Province China

**Keywords:** Anti-epidermal growth factor receptor monoclonal antibodies, Apoptosis, Cervical cancer, Hollow gold nanospheres, Megavoltage X-ray, Radiosensitivity

## Abstract

The study aimed to confirm that anti-epidermal growth factor receptor (EGFR) monoclonal antibody-conjugated hollow gold nanospheres (anti-EGFR/HGNs) can be selectively uptaken by cervical cancer cells and induce its apoptosis when combined with radiotherapy, as a result enhancing radiosensitivity of cervical cancer cells. HGNs with a mean diameter of 54.6 ± 7.11 nm and wall thickness of 5.01 ± 2.23 nm were viewed by transmission electron microscopy (TEM). Cell uptake was assayed by inductively coupled plasma atomic emission spectroscopy (ICP-AES). The cytotoxicity on HeLa cells, which were used in our experiment, was assessed by CCK-8 assay. Cell cycle and apoptosis were examined by an Annexin V-FITC/propidium iodide (PI) kit with flow cytometry (FCM). The expression of several critical apoptosis-related proteins, including Bcl-2, Bax, Bad, and active caspase 3, was tested by western blot analysis. Cells treated by anti-EGFR/HGNs showed an obvious increase in nanoparticle uptake compared to naked HGNs. Anti-EGFR/HGNs combined with radiation resulted in a significant growth inhibition, compared with radiation combined with naked HGNs. Anti-EGFR/HGNs remarkably increased the ratio of HeLa cells in the G2/M phase and induced more apoptosis by an obvious deregulation of Bcl-2 and upregulation of Bax, Bad, and caspase 3 when combined with radiation. Therefore, anti-EGFR/HGNs can increase the targeted uptake of HGNs by HeLa cells and enhance radiocytotoxic targeting of cervical cancer at megavoltage radiation energies.

## Background

Uterine cervical cancer is the second most common malignancy among women worldwide [1]. Despite that numerous advances have been reached in early diagnosis and treatment of cervical cancer in recent years, there is still an overall trend that the mortality is rising. This is partly because some patients continue to present with advanced-stage disease for which conventional therapy is less effective [2]. Radiotherapy is of vital importance for the patients with advanced stage (IIB–IV) which always develop distant metastasis and are unsuitable to be treated with operation, while the outcome is not always satisfactory due to radioresistance. Therefore, novel therapeutic agents are urgently needed to enhance radiosensitivity so as to improve the outcome in these patients. Gold nanoparticles (GNPs) have been considered to have a radiosensitization effect [3] and are widely applied because of their bioinert, nontoxic, readily synthesized, and functionalized characteristics [4]. Among various gold nanostructures, hollow gold nanospheres (HGNs) as a novel class of nanostructures for their small size, spherical shape, hollow interior, and thin but robust wall have won much more attention [5]. The well-established surface chemistry for GNPs allows one to target cells specifically by conjugating different moieties (e.g., antibodies, peptides, and DNAs) to the nanoparticles [6], and the conjugates have good biocompatibility and maintain their original biological activity after conjugation. Previous studies showed that GNPs conjugated with tumor-specific antibodies can lead to an increased concentration of nanoparticles inside cells caused by active targeting of nanoparticles to the tumor site through receptor-mediated endocytosis (RME) [7].

Epidermal growth factor receptor (EGFR) is a cell surface receptor which plays a significant role in signaling pathways, which regulate cell proliferation, angiogenesis, and tumor metastases [8]. Previous studies have demonstrated EGFR to be frequently overexpressed in primary cervical cancer [9–13]. In this study, immunofluorescence staining was adopted to further demonstrate the high expression of EGFR on the cytomembrane of HeLa cells. Anti-EGFR monoclonal antibodies (anti-EGFR) were conjugated to HGNs with stable covalent bonds by bifunctional thiol-containing polyethylene glycol (PEG) to form anti-EGFR/HGNs. The experiment aimed to confirm that anti-EGFR/HGNs can be selectively localized around the cytoplasmic membrane of cervical cancer cells to promote HGN entry into tumor cells by both active and passive targeting. Therefore, anti-EGFR/HGNs will be selectively uptaken by cervical cancer cells and induce their apoptosis, as a result reducing nonspecific injury to normal cells when combined with radiotherapy.

## Methods

### Cell Culture

Human cervical cancer cell line HeLa, obtained from the Gynecological Tumor Laboratory of Qilu Hospital of Shandong University, was used in the experiment, which was cultured in RPMI Medium 1640 (GIBCO, Invitrogen Corporation) supplemented with 10 % fetal bovine serum (FBS) (Kangyuan Biology, China) and 1 % penicillin–streptomycin (Solarbio, Beijing Solarbio Science & Technology, China) in water-jacketed CO_2_ incubators (Thermo Fisher Scientific Forma Series II, USA) at 37 °C with 95 % (*v*/*v*) air and 5 % (*v*/*v*) CO_2_. Cells were used for experiments when they were in the logarithmic growth phase. All study was approved by the ethical review boards and in accordance with the principles of the Declaration of Helsinki.

### Chemicals

Gold (III) chloride trihydrate (HAuCl_4_ · 3H_2_O, G4022-1 g), *N*-(3-dimethylaminopropyl)-*N*-ethylcarbodiimidehydrochloride (EDC), and *N*-hydroxy succinimide (NHS) were obtained from Sigma–Aldrich, USA. Thiol–PEG–carboxyl (SH-PEG-COOH) (Mw = 5000 kDa) was purchased from Shanghai Xibao Medpep Co. Phosphate-buffered solution (PBS) (pH 7.4, 0.1 M) as working buffer solution was prepared with 0.1 M Na_2_HPO_4_, 0.1 M KH_2_PO_4_, and 0.1 M KCl.

### Immunofluorescence Staining

HeLa cells were cultured on glass slides in a 24-well plate and incubated for 24 h. Then, cells were fixed with 4 % paraformaldehyde and blocked with normal goat’s serum for 30 min at room temperature. After thorough washing with PBS, the cells were incubated with primary monoclonal mouse anti-human anti-EGFR antibody (Abcam, MA, USA, 1:200 dilution) overnight at 4 °C and stained with TRITC-conjugated goat anti-mouse IgG (Boster, China, 1:50 dilution) for 1 h. The nuclei were stained by DAPI for 2 min. The fluorescence-labeled EGFR was observed under a fluorescence microscope (Olympus, Japan) and photographs were taken.

### Synthesis of HGNs

Silver colloid was prepared firstly for synthesis of HGNs. One hundred eighty milligrams of silver nitrate was mixed with 100 ml of deionized water under rigorous stirring in a nitrogen atmosphere created by high-purity nitrogen passed into the solution, which was maintained during the reaction. When the reaction solution was rapidly heated to boiling, 1 ml of 1 % sodium citrate was put in and continued to heat for more 30 min. Then, the solution was allowed to cool to room temperature. Galvanic replacement reaction between HAuCl_4_ and silver colloid was adopted to prepare HGNs in an aqueous solution under refluxing condition. Ten milliliters of the silver colloid was put into a 50-ml flask with continuous magnetic stirring and then heated to 60 °C. Meanwhile, an aqueous solution of 5 ml HAuCl_4_ (1 mM, Aldrich, 520918) was slowly added to the flask at a rate of 45 ml/h under magnetic stirring. The solution was heated for 30 min and then concentrated at 2000 × *g* for 5 min. The diameter and morphology of the particles were viewed by transmission electron microscopy (TEM) (JEM-100CX, Japan), and the concentration was tested by inductively coupled plasma atomic emission spectroscopy (ICP-AES) (IRIS INTREPID II XSP).

### Modification of HGNs

Bifunctional SH-PEG-COOH was used as a linker to conjugate antibodies to HGNs, which is in a lyophilized form stored in an argon environment. Firstly, 200 μl of a 2-mM aqueous solution of SH-PEG-COOH was mixed with 200 μl HGNs (10 nM in concentration) to react at 4 °C in the dark for one night. EDC and NHS were added in the solution at room temperature and reacted for half an hour to activate the carboxyl terminal of PEG. Excess EDC and NHS were removed from HGNs by centrifugal separation (2000 × *g*, 5 min). In the second step, 200 μl PEGylated HGNs was mixed with 5 μl mouse anti-human monoclonal anti-EGFR antibody (Abcam, MA, USA, 1 μg/μl) for 2 h at room temperature. Conjugate solutions were centrifuged (2000 × *g*, 5 min) and resuspended in deionized water to remove excess antibody and then stored at 4 °C for future use within 4 days. Addition of 10 % NaCl solution and any visible color change in the colloidal solution can ascertain the successful conjugation of antibodies on HGNs. The final concentration of NaCl in the HGN solution is 0.85 mol/l. The presence of NaCl can cause unconjugated HGNs to aggregate and result in a visible color change from red to purple. Spectrophotometry (Shimadzu UV-2401 PC) was also performed to confirm conjugation of anti-EGFR to HGNs.

### Cytotoxicity Testing

HeLa cells were seeded in a 96-well tissue culture plate, approximately 5 × 10^3^ per well, and incubated overnight. The medium was replaced by a fresh medium containing different concentrations of naked HGNs (0, 1, 3, and 5 nM) and anti-EGFR/HGNs (0, 1, 3, and 5 nM), respectively. To avoid binding and internalization of FBS with HGNs, a FBS-free medium was used. Twenty-four hours later, the medium containing HGNs was removed, the cells were washed twice with PBS, and a new medium with FBS was added. Cells were then incubated at different time intervals (6, 12, 24, and 48 h, respectively), and CCK-8 assay (EnoGene, China) was used to measure cell viability according to the manufacturer’s protocol. Cellular survival rates in response to HGNs were determined by a microplate reader (Bio-Rad Model 680, Richmond, CA, USA) and expressed as the absorbance at 450-nm wavelength.

### Cell Uptake of HGNs and Anti-EGFR/HGNs

A HeLa cell suspension was seeded and cultured in a 25-ml culture flask. Cells were exposed to control, 5 nM naked HGNs, and 5 nM anti-EGFR/HGNs via an FBS-free medium when a confluence of 70 % was reached. After different incubation time intervals (3, 6, 12, 24, and 48 h), cells were washed twice by PBS and collected. Then, cells were resuspended in 5 ml PBS and counted by a hemocytometer. One milliliter of aqua regia (mixture of three parts HCl and one part HNO_3_) was added into each sample to lyse the cells. ICP-AES was used to measure gold content in the lysis solution to further demonstrate and quantify the cell uptake of both naked HGNs and immuno HGNs. Gold has a face-centered cubic (FCC) structure, of which the packing efficiency is 74.05 %. The radius of gold atoms is 134 pm. Assuming that the outer radius of each HGN is *R* nm, the inner radius is *r* nm, and the average gold atom number of each HGN is *n*, according to the following formula: *n* × 4/3 × *π* × 0.134^3^ = 4/3 × *π* × (*R*^3^ − *r*^3^) × 74.05 %, the average number of gold atoms per HGN was obtained by calculation, which is 205. The average molecular weight of HGN = 205 × 197 = 40,385 g/mol. The number of HGNs was calculated according to the gold mass. Then, the number of HGNs uptaken per cell was achieved by the following formula: number of HGNs in the lysis/number of cells.

### Irradiation and Cell Survival Assay

As described before, HeLa cells seeded in 96-well tissue culture plates and incubated overnight were exposed to 5 nM anti-EGFR, 5 nM naked HGNs, or 5 nM anti-EGFR/HGNs for 24 h. Cells were then divided into three groups: one without irradiation and the other two were irradiated by high-energy 6-MV photons, by a medical linear accelerator (Varian 23EX linear accelerator, USA), either with a total dose of 5 or 10 Gy, respectively. Cells treated with nothing served as control. Anti-EGFR antibodies were known to block EGF by binding to the receptor to interfere with EGFR signal transduction, so the cytotoxicity of anti-EGFR antibodies alone and in the presence of irradiation was also evaluated. Three hours after irradiation, cell survival rate was measured by CCK-8 assay.

### Apoptosis Assay

An Annexin V-FITC Apoptosis Detection Kit I (BD Biosciences, USA) was used to detect HeLa cell apoptosis according to the manufacturer’s protocol. Briefly, cells (2 × 10^5^) incubated with 5 nM anti-EGFR/HGNs for 24 h were irradiated with or without X-ray of a dose of 5 Gy. Incubated 3 h after irradiation, the cells were harvested and washed twice with cold PBS and resuspended in 400 μl Annexin V-FITC binding buffer. Cells were stained with 5 μl of Annexin V-FITC and incubated in the dark at 4 °C for 15 min followed by 10 μl propidium iodide (PI) mixed in, and the sample was incubated for 5 min in the same condition. A FACSCalibur flow cytometer (Becton–Dickinson, USA) was used to detect cell apoptosis. This experiment was repeated three times.

### Cell Cycle Analysis

HeLa cells were seeded in six-well dishes, 2 × 10^5^ per well. After incubation overnight, cells were treated with 5 nM naked HGNs and 5 nM anti-EGFR/HGNs respectively for 4 h. Control was treated with medium only. According to the manufacturer’s protocol, cells were collected and fixed overnight in 70 % (*v*/*v*) ethanol at −20 °C. Then, the cells were centrifuged and washed once and resuspended in 200 μl cold PBS. Twenty microliters of RNase was put into each sample to react for 30 min at 37 °C water bath, followed by filtering and staining by 400 μl PI for 1 h at 4 °C in the dark. The samples were tested with a FACSCalibur flow cytometer (Becton–Dickinson, USA). Five thousand cells of each sample were measured, and the data were analyzed by FlowJo software.

### Western Blot Analysis

At 24 h after irradiation, a protein extraction solution (Beyotime, China) with a protease inhibitor was used for protein extraction of HeLa cells, and the protein was quantified with a BCA Protein Assay Kit (Beyotime, China). Samples containing 20 μg protein were loaded and electrophoresed on SDS-polyacrylamide gels (PAGE) and transferred to a PVDF membrane via the Bio-Rad electrotransfer system. Five percent *w*/*v* nonfat dried milk was used to block the membranes for 1 h at room temperature, and then the membranes were incubated with primary rabbit anti-human antibodies specific to Bcl-2, Bax, Bad, and active caspase 3 (Cell Signaling Technology, Beverly, MA, USA; 1:1000 dilution) overnight at 4 °C. Tris-buffered saline with Tween 20 (TBST—20 mM Tris–HCl, pH 7.4; 150 mM NaCl; and 0.05 % Tween 20) was used to wash the membranes. After being incubated in HRP-conjugated goat anti-rabbit secondary antibody (Cell Signaling Technology, Beverly, MA, USA; 1:2000 dilution) for 1 h and washed again, protein bands were visualized by enhanced chemiluminescence (Millipore). GAPDH (Cell Signaling Technology, Beverly, MA, USA; 1:1000 dilution) was used as internal control for comparison and analysis.

### Statistical Analysis

SPSS (SPSS Inc., Chicago, IL, USA) 16.0 software was used to perform statistical analysis. Experimental values were determined in triplicate and expressed as means and standard errors (SE). The Student *t* test and one-way analysis of variance (ANOVA) were adopted. *P* < 0.05 was considered statistically significant.

## Results and Discussion

### Immunofluorescence Assay

**Fig. 1:**
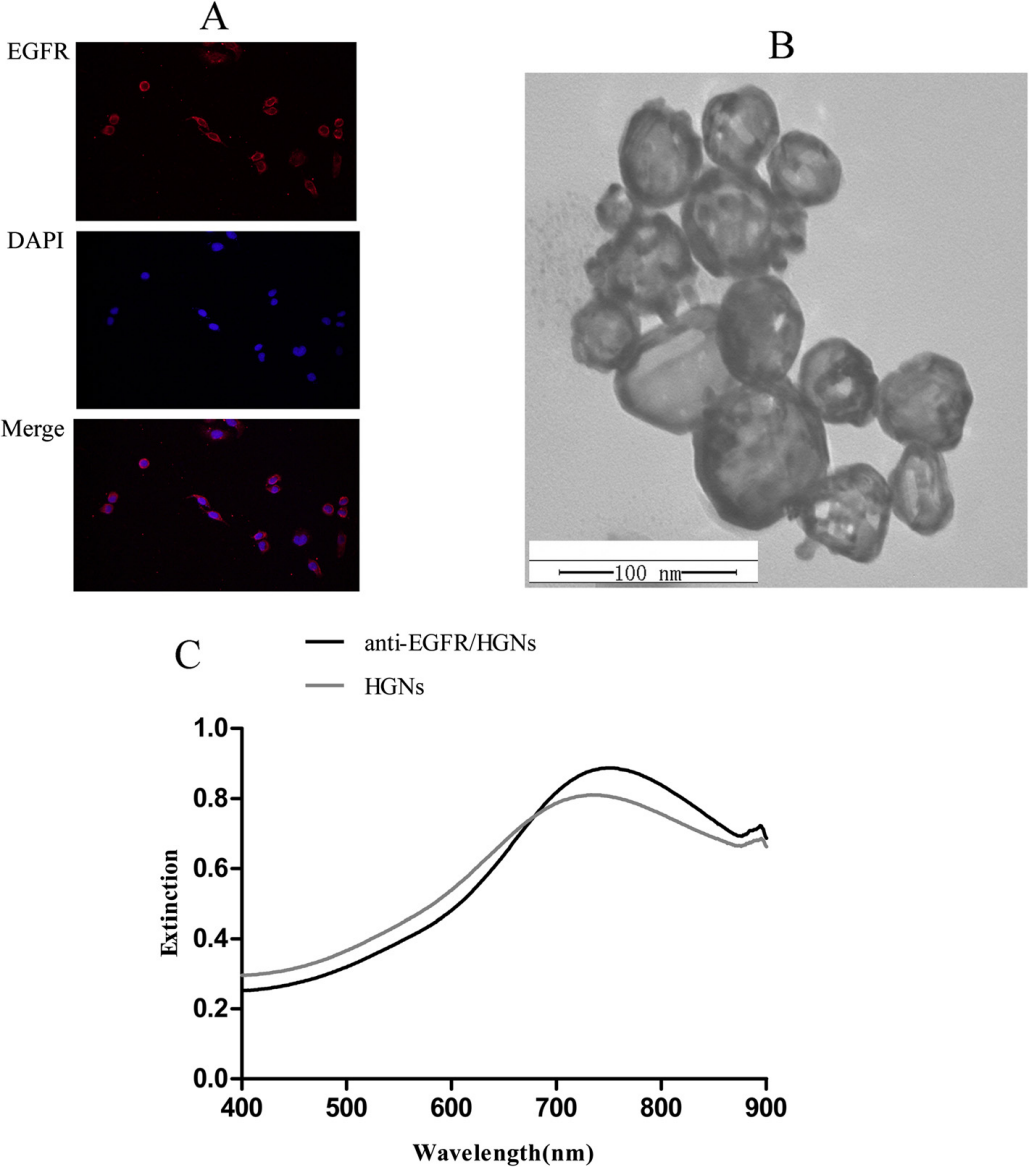
**a** A layer of red fluorescent staining on the cytomembrane of HeLa cells (original magnification × 400). **b** A TEM picture of HGNs. **c** UV–vis extinction of naked HGNs and anti-EGFR/HGNs

From Fig. 1a, a layer of red fluorescent staining was shown on the cytomembrane of HeLa cells, and this can serve as an evidence of the high expression of EGFR.

### Synthesis and Characterization of HGNs

HGNs have been synthesized and characterized, which were with a mean diameter of 54.6 ± 7.11 nm and a wall thickness of 5.01 ± 2.23 nm, calculated by measurements obtained from TEM. From Fig. 1b, the hollow interior and thin shell of HGNs can be shown clearly. The surface plasma resonance (SPR) peak for HGNs was approximately at 733 nm based on the UV–vis extinction spectrum as shown in Fig. 1c.

### Conjugation of Anti-EGFR to HGNs

Conjugation of anti-EGFR to HGNs was confirmed by spectrophotometry. The extinction spectrum of HGNs both before and after conjugation with anti-EGFR was measured and shown in Fig. 1c, and a characteristic redshift (≈20 nm) of the peak of SPR, which is typical of protein binding on the surface of GNPs, demonstrates successful conjugation of anti-EGFR on HGNs. After addition of 10 % NaCl solution to the samples, we did not see any visible color change in the anti-EGFR/HGNs compared to the control, while the color turned to purple in the naked-HGN group. This could serve as another evidence of successful conjugation of anti-EGFR on HGNs.

### Cytotoxicity of Naked HGNs and Anti-EGFR/HGNs

**Fig. 2:**
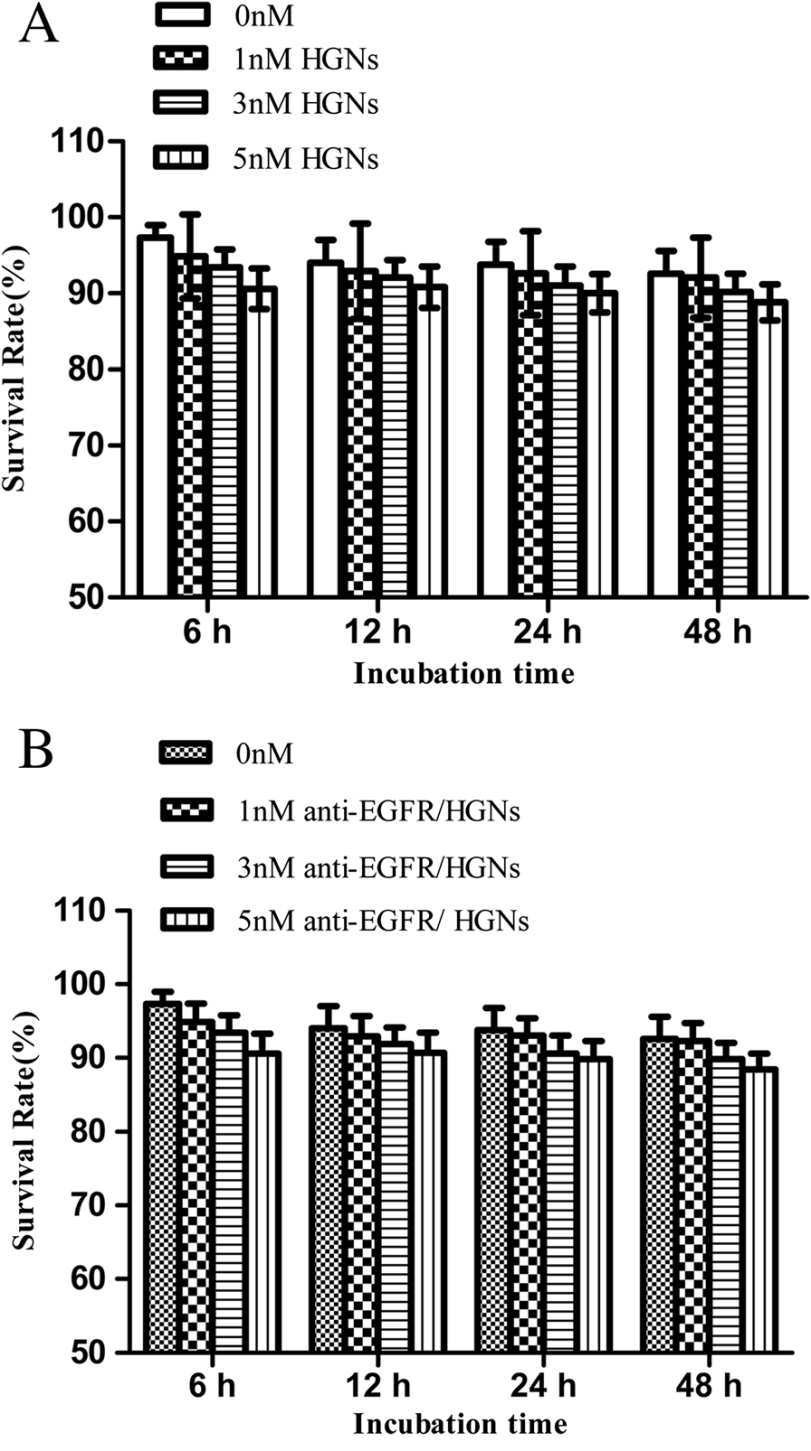
Cytotoxicity of HGNs. Survival rates of HeLa cells treated by (**a**) naked HGNs and (**b**) anti-EGFR/HGNs with different concentrations (0, 1, 3, and 5 nM) at 6, 12, 24, and 48 h, respectively, measured by CCK-8 assay

Different concentrations of naked HGNs (0, 1, 3, and 5 nM) and anti-EGFR/HGNs (0, 1, 3, and 5 nM) were incubated with cells separately, and cytotoxicity of the nanoparticles without radiation was measured by CCK-8 assay. Fig. 2a shows the cell survival rates for four groups with 0, 1, 3, and 5 nM naked HGNs were 97.32, 94.86, 93.40, and 90.60 % respectively at 6 h (*P* > 0.05) and 92.60, 92.06, 90.2, and 88.8 % respectively at 48 h (*P* > 0.05). As for anti-EGFR/HGNs, the survival rates for cells were 97.32, 94.86, 93.4, and 90.6 % respectively at 6 h (*P* > 0.05) and 92.6, 92.26, 89.8, and 88.4 % respectively at 48 h (*P* > 0.05) as shown in Fig. 2b. These results in cell viability analysis indicated neither naked HGNs (1, 3, and 5 nM) nor anti-EGFR/HGNs (1, 3, and 5 nM) induce remarkable cytotoxicity on HeLa cells. Survival rate = (average OD450 nm of the treated group/average OD450 nm of the control group) × 100 %.

### Uptake of Naked HGNs and Anti-EGFR/HGNs

**Fig. 3:**
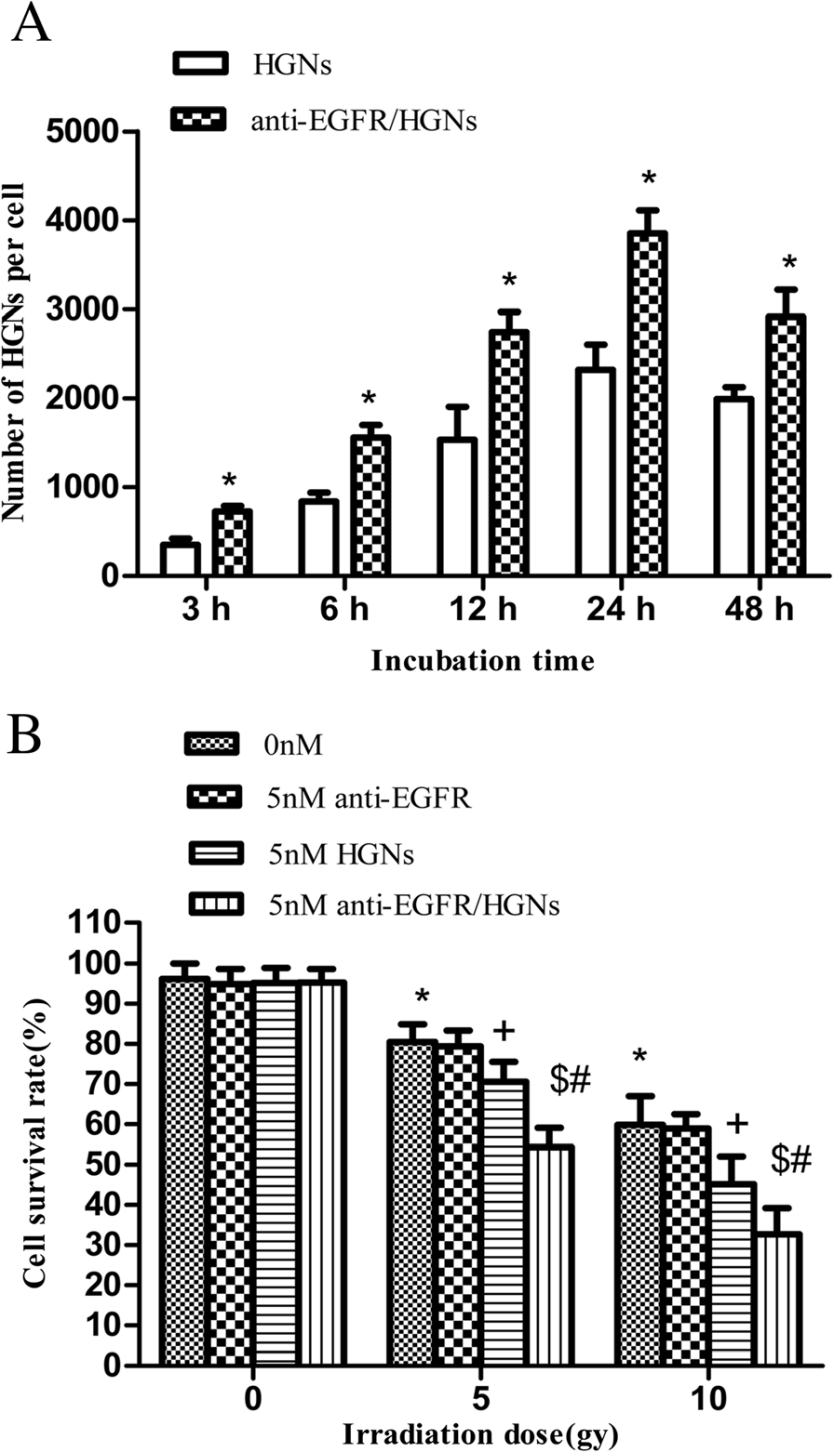
**a** Uptake of naked HGNs vs anti-EGFR/HGNs per cell measured by ICP-AES. *P < 0.05 (naked HGNs vs anti-EGFR/HGNs). **b** Enhanced radiation susceptibility. The viability of cells which were incubated with nothing, 5 nM naked HGNs, 5 nM anti-EGFR, or 5 nM anti-EGFR/HGNs was measured by CCK-8 assay at 3 h after being irradiated by 0, 5, and 10 Gy, respectively. *P < 0.05 (X-ray vs control); ^#^ *P <* 0.05 (anti-EGFR/HGNs vs naked HGNs); ^+^ *P* < 0.05 (naked HGNs vs control); ^$^ *P* < 0.05 (anti-EGFR/HGNs vs control)

ICP-AES was used to quantify the numbers of HGNs uptaken by HeLa cells. Fig. 3a shows the average number of nanoparticles uptaken by each cell at individual time points (3–48 h). The uptake of both naked HGNs and anti-EGFR/HGNs increased with incubation time during the first 24 h, and the peak uptake concentrations for both were achieved at 24 h. Much more anti-EGFR/HGNs were absorbed by HeLa cells than naked HGNs at each time interval (*P* < 0.05).

### Enhanced Radiosensitivity of HeLa Cells

The results showed both naked HGNs and anti-EGFR/HGNs enhanced radiation sensitivity of HeLa cells compared to groups treated with X-ray radiation alone, while conjugates subject to X-ray radiation reduced survival rate more remarkably. For example, irradiation alone (5 Gy) induced a survival rate of 80.4 % for HeLa cells, while cell survival rates of groups treated by 5 Gy of irradiation combined with 5 nM naked HGNs or 5 nM anti-EGFR/HGNs were reduced to 70.54 and 54.28 %, respectively (*P* < 0.05), as shown in Fig. 3b. As for groups irradiated by 10 Gy, a significant decrease in survival rate was observed; survival rates were 59.8 % for X-ray alone and 45.18 and 32.66 % for naked HGNs and anti-EGFR/HGNs, respectively (*P* < 0.05). When it comes to the cytotoxicity of anti-EGFR alone and in the presence of irradiation, results showed that survival rates of groups treated by 5 nM anti-EGFR combined with 0, 5, or 10 Gy were 94.86, 79.3, and 58.9 %, respectively, approximate to groups exposed to 0 nM (*P* > 0.05). So, the synergic action of anti-EGFR and HGNs was mainly due to HGNs and the effect of EGFR was subtle if any in this experiment.

### Apoptosis Detection

**Fig. 4:**
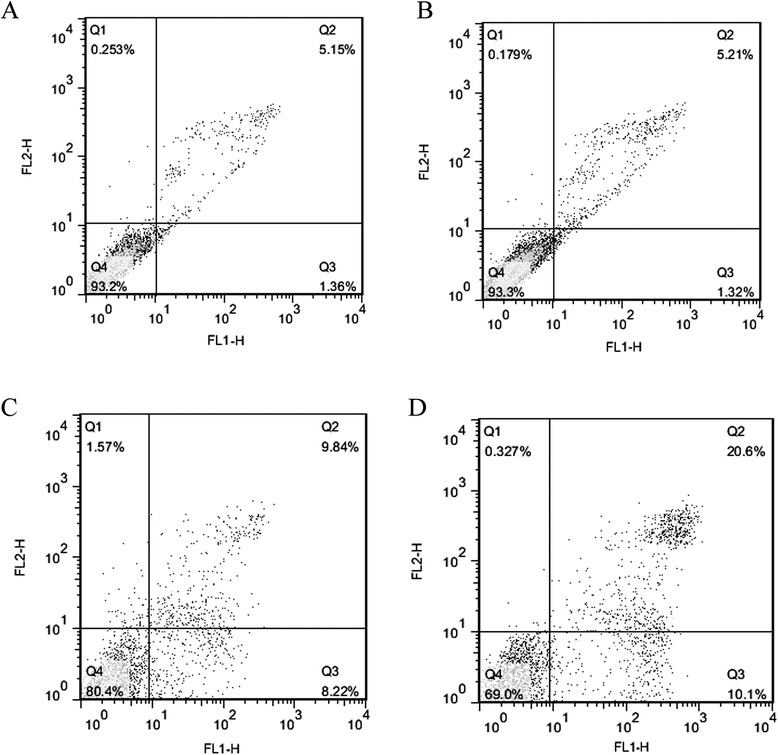
Analysis of apoptosis in HeLa cells with Annexin V and PI staining. Anti-EGFR/HGNs subject to X-ray irradiation induced a significant increase in apoptosis. **a** Control, (**b**) anti-EGFR/HGNs alone, (**c**) X-ray alone, and (**d**) anti-EGFR/HGNs *+* X-ray

Cell apoptosis has been demonstrated to play a considerable part in cell growth, so we further assessed whether functional HGNs combined with X-ray radiation induced more apoptosis. Cells in the right lower and upper quadrants are considered in early- and late-stage apoptosis, respectively, and those in the left upper quadrants are considered as dead cells. The sum of proportion of right lower and upper quadrants represents the apoptotic rate. As shown in Fig. 4a, b, exposure to anti-EGFR/HGNs without X-ray did not induce significant increases in the apoptosis (6.53 ± 1.6 %) of HeLa cells compared to controls (6.51 ± 1.0 %) (*P* > 0.05). X-ray irradiation increased the apoptosis (18.06 ± 1.3 %) of HeLa cells compared to controls (6.51 ± 1.0 %) (*P* < 0.05) (Fig. 4a, c). Anti-EGFR/HGNs combined with X-ray irradiation induced more apoptosis (30.7 ± 2.2 %) compared to irradiation alone (18.06 ± 1.3 %) (*P* < 0.05) (Fig. 4c, d). These data indicate increased cell apoptosis is one of the mechanisms of the enhanced radiosensitivity of anti-EGFR/HGNs.

### Cell Cycle Assay

**Fig. 5:**
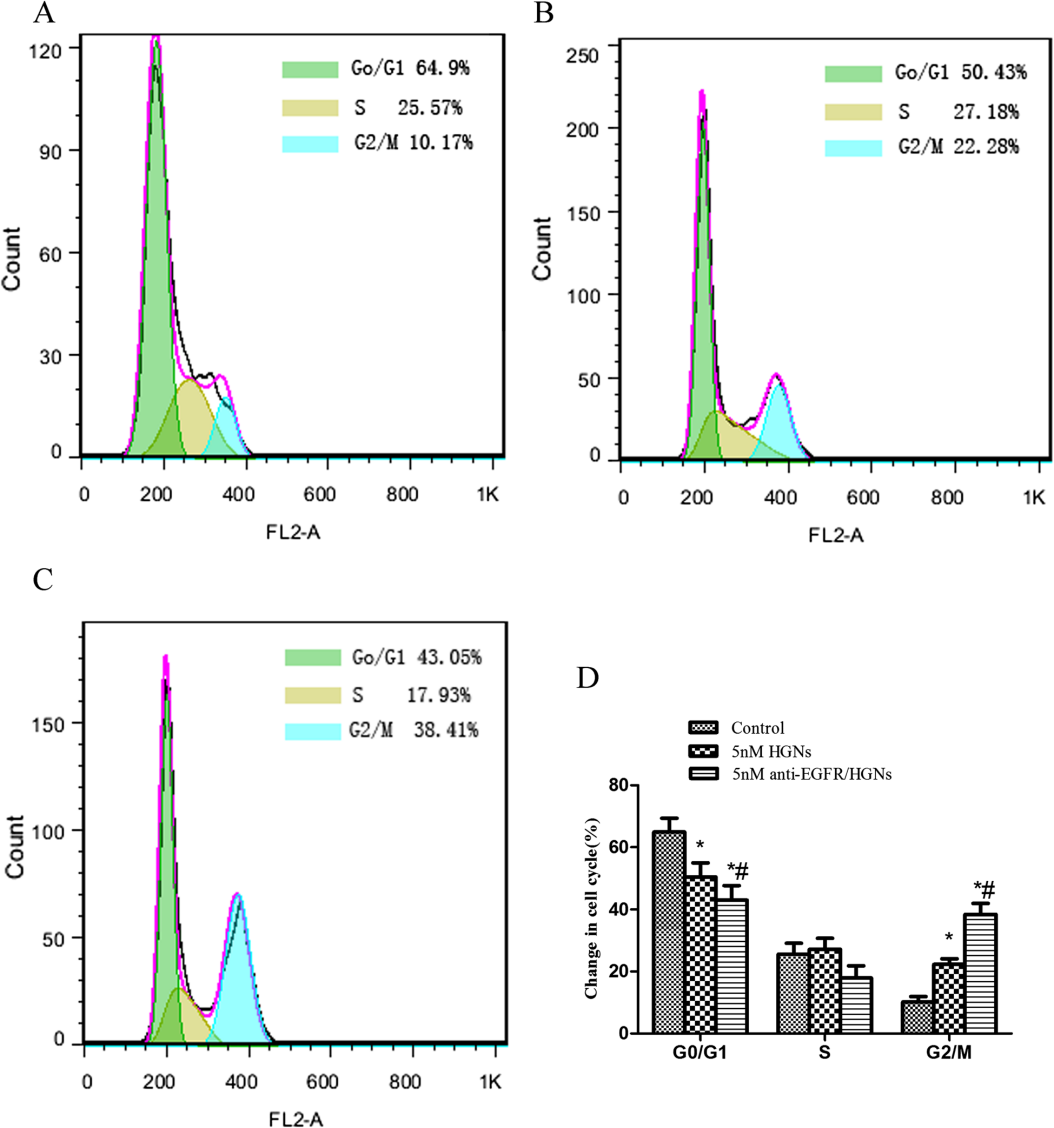
Flow cytometric analysis of cell cycle in HeLa cells. **a** Control, (**b**) 5 nM naked HGNs, (**c**) 5 nM anti-EGFR/HGNs, and (**d**) comparison of changes of the cell cycle. *P < 0.05 (HGNs vs control); ^#^ *P <* 0.05 (anti-EGFR/HGNs vs naked HGNs)

HeLa cells were treated with 5 nM naked HGNs and 5 nM anti-EGFR/HGNs for 4 h, and cells in the G2/M phase increased obviously while cells in the G0/G1 phase decreased compared with the control (Fig. 5). Of the untreated control cells, 10.17 % were in the G2/M phase, while naked HGNs and anti-EGFR/HGNs increased the fraction of cells to 22.28 and 38.41 %, respectively (*P* < 0.05). For the G0/G1 phase, 64.90 % for the control was decreased to 50.43 % and 43.05 % for cells treated with 5 nM naked HGNs and 5 nM anti-EGFR/HGNs, respectively (*P* < 0.05). Cells were arrested at G2/M, the most sensitive phase to radiation of the cell cycle, and thereby enhanced radiation sensitivity of HeLa cells.

### Expression of Apoptosis-Related Proteins

**Fig. 6:**
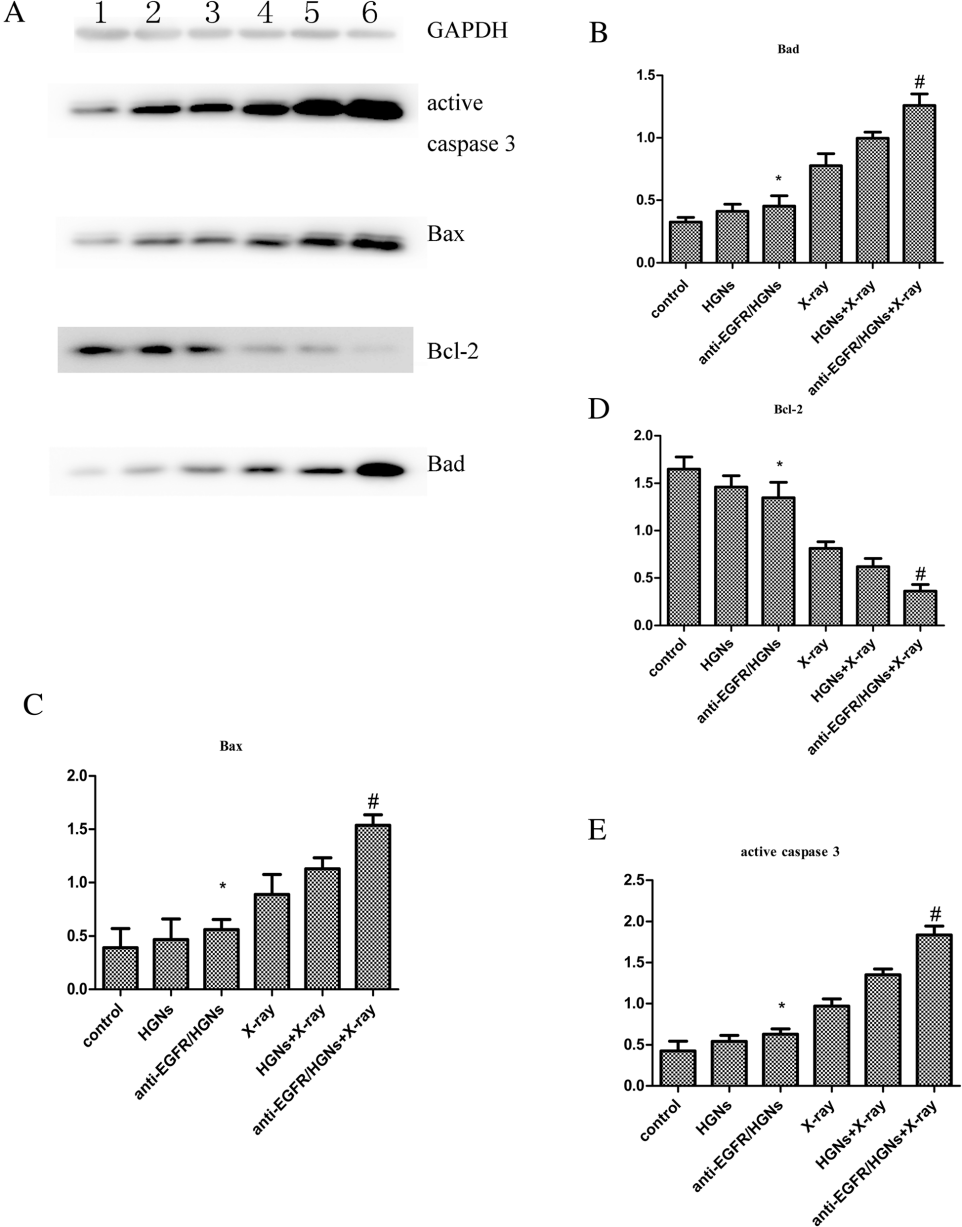
Western blot of protein expression of Bcl-2, Bax, Bad, and active caspase 3. **a** *Lane 1* indicates control, *lane 2* naked HGNs, *lane 3* anti-EGFR/HGNs, *lane 4* X-ray alone, *lane 5* naked HGNs + X-ray, and *lane 6* anti-EGFR/HGNs + X-ray. The diagrams show the ratio of intensities of the Bad (**b**), Bax (**c**), Bcl-2 (**d**), and active caspase 3 (**e**) bands to the GAPDH band. *P > 0.05 (anti-EGFR/HGNs vs control); ^#^ *P <* 0.05 (anti-EGFR/HGNs + X-ray vs X-ray)

The expression of several critical apoptosis-related proteins was tested to further explore the molecular mechanism involved in increased apoptosis caused by anti-EGFR/HGNs, including Bcl-2, Bax, Bad, and active caspase 3. A significant upregulation of Bax, Bad, and active caspase 3 was shown in western blot analysis for the sample treated with anti-EGFR/HGNs combined with radiation (*P* < 0.05), while the expression of Bcl-2 was obviously decreased in this group (*P* < 0.05) (Fig. 6). The result demonstrated that anti-EGFR/HGNs can enhance radiosensitivity and promote cancer cell apoptosis by regulating the expression of the Bcl-2 family of proteins and active caspase 3.

Human cervical cancer is the second most common malignancy among women worldwide [1]. About 500,000 new cases of cervical cancer are diagnosed each year, resulting in 250,000 deaths [14]. The 5-year survival rate for patients with stage III is 25 to 35 %, while for stage IV, it is 15 % or less [15]. Despite availability of screening, cervical cancer is still the third leading cause of cancer-related mortality [16]. Radiotherapy as a conventional regimen is not always satisfactory for patients with high-risk and advanced cervical cancer because of the onset of radioresistance. Therefore, to enhance the radiosensitivity is of vital importance for the radiation therapy of cervical cancer.

EGFR overexpression has been reported in many experimental cell lines and human carcinomas, such as head and neck cancer [17], esophageal cancer [18], gastric cancer [19], and primary cervical cancer [9–13]. As for uterine cervical cancer, a large amount of evidences demonstrate that elevated levels of EGFR are closely relevant to a more aggressive biological behavior and further to poor prognosis in cervical cancer patients clinically [20], making EGFR an attractive candidate for anti-cancer therapy. Immunofluorescence staining was adopted to further demonstrate high expression of EGFR on the membrane of HeLa cells. Recent reports of EGFR targeting strategies have involved use of anti-EGFR antibodies to block EGF by binding to the receptor or as a targeting ligand for the delivery of therapeutic agents [21]. However, the blocking effect of anti-EGFR antibodies was subtle if any in our study, and they were mainly used as a targeting ligand for the delivery of HGNs. EGFR-targeted therapy has recently been applied as a new therapeutic strategy in a variety of malignant tumors for its apparent correlation with radiotherapy [22].

GNPs are widely applied because of their biocompatibility, nontoxic, readily synthesized, and functionalized characteristics [4]. Geng et al. investigated Glu-GNPs-enhanced target cytotoxicity of radiation on ovarian cancer cells and demonstrated that GNPs have remarkable potential to enhance radiotherapy [23]. Among various gold nanostructures, HGNs as a novel class of nanostructures for their small size, spherical shape, hollow interior, and thin but robust wall have won much more attention. Liu et al. investigated HGNs for targeted photothermal destruction of cervical cancer cells which showed a strong absorption peak at 780 nm and could efficiently convert light to heat to destroy tumor cells when exposed to near-infrared (NIR) laser light [5]. We tried to explore whether HGNs have potential in the effect of radiosensitization of cervical cancer cells.

In theory, the size of gold nanoparticles should be large enough in case of rapid leakage from tissue into blood capillaries; meanwhile, it should be small enough to escape capture by macrophages. Chithrani et al. have studied the uptake of 14, 50, and 74 nm citrate-coated GNPs in HeLa cells and found particles of 50 nm were uptaken most efficiently by tumor cells [24]. The size of HGNs in our study was 54.6 ± 7.11 nm and the wall thickness was 5.01 ± 2.23 nm, as determined by TEM, which was close to the optimal condition.

Various approaches for the delivery of nanoparticles to targeted tumor sites have been developed [25]. GNPs can passively target tumor sites by enhanced permeability and retention (EPR) effect in neoplasm lesions [26]. Earlier studies have investigated various surface modifications of nanoparticles with active ligands to achieve further accumulation of nanoparticles in tumor cells, which is commonly referred to as active targeting and can remarkably enhance efficiency of specific endocytosis. Meanwhile the combinement of active targeting and passive targeting can achieve the maximum localization of nanoparticles in malignant tissues [27, 28]. In our study, HGNs were conjugated to monoclonal anti-EGFR antibodies via bifunctional thiol-containing PEG and the conjugates were allowed to interact with cancer cells. The approximate 20-nm redshift in the SPR peak of HGNs after conjugation is due to alterations in the local refractive index owing to the presence of a layer of antibodies on their surface. This redshift is typical if proteins are bound on the surface of GNPs and serves as an evidence of successful conjugation [29].

A major concern of HGNs in biological applications is the toxicity. The toxicity of both naked HGNs and anti-EGFR/HGNs on HeLa cells was tested, and neither naked HGNs nor anti-EGFR/HGNs induced remarkable cytotoxicity with the increase of gold concentration (1–5 nM) and incubation time (6–48 h), while a decrease in cell viability was observed (*P* > 0.05). A recent study showed that GNPs modified with a variety of molecules are not toxic to human cells essentially, let alone acute cytotoxicity [30].

Recent studies that have investigated GNPs as a radiosensitizer usually focused on kV radiosensitization, while GNPs have also been reported to enhance radiosensitivity with MV photons and electrons in breast cancer cells [31]. With the development of radiological technology, MV radiation has gradually replaced low-energy radiation in clinical radiotherapy nowadays [32]. MV X-rays were adopted to demonstrate the radiosensitization by anti-EGFR/HGNs for the radiotherapy of cervical cancer. Significantly enhanced radiosensitivity by anti-EGFR/HGNs of HeLa cells irradiated at 6-MV X-rays was confirmed in our study.

About the mechanism of enhanced radiosensitivity, Turner et al. reported metallic materials may arrest cells at the G2/M phase, the most radiosensitive phase of the cell cycle [33]. Zhang et al. reported GNPs trigger activation of the CDK kinases, leading to accumulation in the G2/M phase of cancer cells [34], and as a result disproportionately increase the sensitivity toward radiation. GNPs have been observed to enhance radiosensitivity by regulating cell cycle and increasing the level of reactive oxygen species (ROS) [35]. In this study, after treatment with anti-EGFR/HGNs, HeLa cells which uptook more HGNs were arrested at the G2/M and were more sensitive to radiotherapy.

Apoptosis as an ordered cellular process is of vital importance in regulating cell death [36] so that activation of apoptosis is widely considered as an anticancer strategy. Dual staining of cells with Annexin V-FITC and PI was adopted to quantitatively measure cell apoptosis. A dramatic increase of apoptosis of HeLa cells was observed in the group treated by a combination of anti-EGFR/HGNs and 6-MV X-rays, as shown by FCM.

In addition, anti-EGFR/HGNs also played a role by regulating expression of several critical apoptosis-related proteins, such as the Bcl-2 family proteins, and active caspase 3. The Bcl-2 family of proteins, including Bcl-2, Bax, and Bad, are critical regulators of apoptosis. Bcl-2 plays anti-apoptotic roles and overexpression of Bcl-2 is relevant to the onset of cancer. Both Bax and Bad have a pro-apoptotic effect. Caspase 3 plays pivotal roles in the extrinsic pathway of apoptosis and is considered as the most important performer of apoptosis in the caspase family [37]. The active caspase 3 stirs up cell death by cleaving death substrate PARP into two fragments [38]. Pozo-Guisado et al. observed the expression of caspase 3 may contribute to death of breast cancer cells [39]. According to our experiment, a significantly increased expression of Bax, Bad, and active caspase 3 and decreased expression of Bcl-2 were shown in the anti-EGFR/HGNs plus radiation group, compared with radiation alone. Therefore, we can get the conclusion that anti-EGFR/HGNs may increase apoptosis of HeLa cells by destroying the balance between anti-apoptotic and pro-apoptotic proteins and activating caspase 3.

## Conclusions

The study demonstrated that anti-EGFR/HGNs significantly increase the cytotoxicity of 6-MV X-rays on HeLa cells, and anti-EGFR/HGNs combined with radiotherapy may possess a large therapeutic potential in cervical cancer. We can also conclude that anti-EGFR/HGNs may be involved in the increased apoptosis of HeLa cells by regulating the expression of Bcl-2 family proteins and activating caspase 3. In addition, anti-EGFR/HGNs manipulate the cancer cell cycle to enhance radiation susceptibility. A large amount of studies still remain to be done: investigating molecular mechanisms governing enhanced radiation cytotoxicity and the radiosensitizing functions of anti-EGFR/HGNs in animal models.

## References

[1] Jones SB (1999). Cancer in the developing world: a call to action. BMJ.

[2] Iida K, Nakayama K, Rahman MT (2011). EGFR gene amplification is related to adverse clinical outcomes in cervical squamous cell carcinoma, making the EGFR pathway a novel therapeutic target. Brit J Cancer.

[3] Hainfeld JF, Slatkin DN, Smilowitz HM (2004). The use of gold nanoparticles to enhance radiotherapy in mice. Phys Med Biol.

[4] Lewinski N, Colvin V, Drezek R (2008). Cytotoxicity of nanoparticles. Small.

[5] Liu T, Tian J, Chen Z (2014). Anti-TROP2 conjugated hollow gold nanospheres as a novel nanostructure for targeted photothermal destruction of cervical cancer cells. Nanotechnology.

[6] Au L, Zheng D, Zhou F, Li Z, Li X, Xia Y (2008). A quantitative study on the photothermal effect of immuno gold nanocages targeted to breast cancer cells. ACS Nano.

[7] Lu W, Zhang G, Zhang R (2010). Tumor site-specific silencing of NF-kappaB p65 by targeted hollow gold nanosphere-mediated photothermal transfection. Cancer Res.

[8] Waksal HW (1999). Role of an anti-epidermal growth factor receptor in treating cancer. Cancer Metastasis Rev.

[9] Pfeiffer D, Stellwag B, Pfeiffer A, Borlinghaus P, Meier W, Scheidel P (1989). Clinical implications of the epidermal growth factor receptor in the squamous cell carcinoma of the uterine cervix. Gynecol Oncol.

[10] Gaffney DK, Haslam D, Tsodikov A (2003). Epidermal growth factor receptor (EGFR) and vascular endothelial growth factor (VEGF) negatively affect overall survival in carcinoma of the cervix treated with radiotherapy. Int J Radiat Oncol Biol Phys.

[11] Ngan HY, Cheung AN, Liu SS, Cheng DK, Ng TY, Wong LC (2001). Abnormal expression of epidermal growth factor receptor and c-erbB2 in squamous cell carcinoma of the cervix: correlation with human papillomavirus and prognosis. Tumour Biol.

[12] Scambia G, Ferrandina G, Distefano M, D’Agostino G, Benedetti-Panici P, Mancuso S (1998). Epidermal growth factor receptor (EGFR) is not related to the prognosis of cervical cancer. Cancer Lett.

[13] Kristensen GB, Holm R, Abeler VM, Trope CG (1996). Evaluation of the prognostic significance of cathepsin D, epidermal growth factor receptor, and c-erbB-2 in early cervical squamous cell carcinoma. An immunohistochemical study. Cancer.

[14] Narayanan B (2011). Epidermal growth factor-stimulated human cervical cancer cell growth is associated with EGFR and cyclin D1 activation, independent of COX-2 expression levels. Int J Oncol.

[15] Lilic V, Lilic G, Filipovic S, Milosevic J, Tasic M, Stojiljkovic M (2009). Modern treatment of invasive carcinoma of the uterine cervix. J BUON.

[16] Rodriguez VS, Diaz-Caneja PC, Cervera GJ (2011). Current opinion in cervix carcinoma. Clin Transl Oncol.

[17] Santini J, Formento JL, Francoual M (1991). Characterization, quantification, and potential clinical value of the epidermal growth factor receptor in head and neck squamous cell carcinomas. Head Neck.

[18] Ozawa S, Ueda M, Ando N, Shimizu N, Abe O (1989). Prognostic significance of epidermal growth factor receptor in esophageal squamous cell carcinomas. Cancer.

[19] Yasui W, Hata J, Yokozaki H (1988). Interaction between epidermal growth factor and its receptor in progression of human gastric carcinoma. Int J Cancer.

[20] Kersemaekers AM, Fleuren GJ, Kenter GG (1999). Oncogene alterations in carcinomas of the uterine cervix: overexpression of the epidermal growth factor receptor is associated with poor prognosis. Clin Cancer Res.

[21] Kao H, Lin Y, Chen C (2013). Evaluation of EGFR-targeted radioimmuno-gold-nanoparticles as a theranostic agent in a tumor animal model. Bioorg Med Chem Lett.

[22] West CM, Joseph L, Bhana S (2008). Epidermal growth factor receptor-targeted therapy. Br J Radiol.

[23] Geng F, Song K, Xing JZ (2011). Thio-glucose bound gold nanoparticles enhance radio-cytotoxic targeting of ovarian cancer. Nanotechnology.

[24] Chithrani BD, Ghazani AA, Chan WC (2006). Determining the size and shape dependence of gold nanoparticle uptake into mammalian cells. Nano Lett.

[25] Huang X, El-Sayed IH, Qian W, El-Sayed MA (2006). Cancer cell imaging and photothermal therapy in the near-infrared region by using gold nanorods. J Am Chem Soc.

[26] Iyer AK, Khaled G, Fang J, Maeda H (2006). Exploiting the enhanced permeability and retention effect for tumor targeting. Drug Discov Today.

[27] Conde J, Doria G, Baptista P (2012). Noble metal nanoparticles applications in cancer. J Drug Deliv.

[28] Ghosh P, Han G, De M, Kim CK, Rotello VM (2008). Gold nanoparticles in delivery applications. Adv Drug Deliv Rev.

[29] Baykul T, Yilmaz HH, Aydin U, Aydin MA, Aksoy M, Yildirim D (2010). Early diagnosis of oral cancer. J Int Med Res.

[30] Connor EE, Mwamuka J, Gole A, Murphy CJ, Wyatt MD (2005). Gold nanoparticles are taken up by human cells but do not cause acute cytotoxicity. Small.

[31] Jain S, Coulter JA, Hounsell AR (2011). Cell-specific radiosensitization by gold nanoparticles at megavoltage radiation energies. Int J Radiat Oncol Biol Phys.

[32] Wang C, Li X, Wang Y, Liu Z, Fu L, Hu L (2013). Enhancement of radiation effect and increase of apoptosis in lung cancer cells by thio-glucose-bound gold nanoparticles at megavoltage radiation energies. J Nanoparticle Res.

[33] Turner J, Koumenis C, Kute TE (2005). Tachpyridine, a metal chelator, induces G2 cell-cycle arrest, activates checkpoint kinases, and sensitizes cells to ionizing radiation. Blood.

[34] Zhang X, Xing JZ, Chen J (2008). Enhanced radiation sensitivity in prostate cancer by gold-nanoparticles. Clin Invest Med.

[35] Roa W, Zhang X, Guo L (2009). Gold nanoparticle sensitize radiotherapy of prostate cancer cells by regulation of the cell cycle. Nanotechnology.

[36] Lagadic-Gossmann D, Huc L, Lecureur V (2004). Alterations of intracellular pH homeostasis in apoptosis: origins and roles. Cell Death Differ.

[37] Wong RS (2011). Apoptosis in cancer: from pathogenesis to treatment. J Exp Clin Cancer Res.

[38] Li X, Zhang Q, Cai L (2009). Inhibitor of growth 4 induces apoptosis in human lung adenocarcinoma cell line A549 via Bcl-2 family proteins and mitochondria apoptosis pathway. J Cancer Res Clin Oncol.

[39] Pozo-Guisado E, Merino JM, Mulero-Navarro S (2005). Resveratrol-induced apoptosis in MCF-7 human breast cancer cells involves a caspase-independent mechanism with downregulation of Bcl-2 and NF-kappaB. Int J Cancer.

